# Detection and Characterization of ESBL‐Producing and Carbapenem‐Resistant *Klebsiella pneumoniae* in Ornamental Birds and Their Surrounding Environments

**DOI:** 10.1002/mbo3.70348

**Published:** 2026-06-23

**Authors:** Md Abdullah Evna Hasan, Md. Liton Rana, Md Saiful Islam, Sadia Afrin Punom, Md. Tabeer Hossain Antor, Diruba Akter Jany, Naeem Ahammed Ibrahim Fahim, S M Abu Sama Al Faruquee, Md. Raisul Islam, Zuhayr Bakhtiyar, Anindita Ash Prome, Md. Monirul Islam, Saifur Rahman, Mohammad Ferdousur Rahman Khan, Md. Tanvir Rahman

**Affiliations:** ^1^ Department of Microbiology and Hygiene, Faculty of Veterinary Science Bangladesh Agricultural University Mymensingh Bangladesh; ^2^ National Engineering Research Center of Industrial Wastewater Detoxication and Resource Recovery, Research Center for Eco‐Environmental Sciences Chinese Academy of Sciences Beijing China; ^3^ Department of Animal Science University of California ‐ Davis Davis California USA

**Keywords:** biofilms, multi‐drug resistant, ornamental birds, public health, virulence

## Abstract

Extended‐spectrum beta‐lactamase‐producing *Klebsiella pneumoniae* (ESBL‐KP) and carbapenem‐resistant *Klebsiella pneumoniae* (CRKP) represent a global health risk, with ornamental birds serving as potential reservoirs for their dissemination. This study aimed to isolate and characterize ESBL‐KP and CRKP from ornamental birds and their surrounding environments. A total of 112 samples, including equal numbers of feces, cage swab, cage water, cage feed, store feed, handler's hand swab, nasal swab, and air samples, were collected equally from both households and pet bird shops. Overall, 52.68% (59/112, CI_95_: 0.435–0.6168) isolates were confirmed as positive, where a higher occurrence was observed in pet store samples (32/56, 57.14%) than in household samples (27/56, 48.21%). In the disk diffusion test, 79.67% (47/59, CI_95_: 0.6773–0.8796) isolates were identified as multidrug‐resistant, with high resistance to imipenem and cefotaxime (both 66.10%), meropenem and ceftriaxone (each 59.02%). The occurrence of ESBL‐producing isolates was higher in shop samples (26/32, 81.25%) than in household samples (12/27, 44.44%). In the Congo Red assay, 76.28% (45/59, CI_95_: 0.6403–0.8531) isolates were identified as strong biofilm producers. Resistance genes, *bla*_CTX‐M_ (92.10%), *bla*_TEM_ (84.21%), *bla*_SHV_ (78.95%), *bla*_OXA‐51_ (51.42%), and *bla*_NDM_ (34.29%), conferring resistance to beta‐lactams and carbapenems were detected in the *K. pneumoniae* isolates, along with virulence genes, *uge* (100%), *entB* (83.05%), *mrkD* (81.36%), and *kfu* (30.51%). As a first report from Bangladesh, this study highlights the widespread occurrence of ESBL‐KP, CRKP and biofilm‐producing *K. pneumoniae* in ornamental birds, emphasizing the urgent need for a One Health approach to prevent associated zoonotic and environmental risks.

## Graphical abstract

Multidrug‐resistant ESBL‐ and carbapenem‐resistant *Klebsiella pneumoniae* were identified from ornamental birds, their handlers and surrounding environments, carrying key virulence and resistant genes, highlighting potential One Health transmission risks between animals, humans and the associated environments.

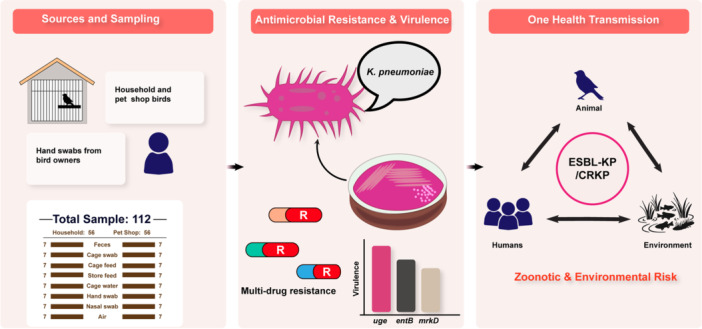

## 1 Introduction

Antimicrobial resistance (AMR) has become one of the most concerning global health issues of the 21st century. The rapid spread of resistant pathogens has rendered many commonly used antibiotics ineffective, which leads to increased treatment failure, morbidity, mortality, and medical costs worldwide, directly causing an estimated 1.27 million deaths in 2019 and projected to cause nearly 1.91 million deaths annually by 2050 (Murray et al. 2022). As a consequence, infectious diseases, particularly those caused by multidrug‐resistant (MDR) bacteria, continue to pose a major global health concern as therapeutic options become increasingly limited. In response to this growing crisis, increasing attention has been given to strategies targeting bacterial virulence mechanisms such as biofilm formation and adhesion, along with bacteriophage therapy, probiotics, and plant‐derived antimicrobials, as potential complements or substitutes to conventional antibiotics (Koo et al. 2017; El Haddad et al. 2019; Hindieh et al. 2025).

Among clinically important pathogens contributing significantly to this burden, *Klebsiella pneumoniae* is a leading cause of multidrug resistance. *K. pneumoniae* is a Gram‐negative, non‐motile, rod‐shaped bacterium belonging to the family Enterobacteriaceae. They are facultative anaerobes and can withstand both aerobic and anaerobic environments (Brisse et al. 1946). It is the most clinically relevant species, causing infections in the liver, bloodstream, respiratory tract, and urinary tract, especially in immunocompromised (Asokan et al. 2025a; Keynan and Rubinstein 2007). It has been listed by the World Health Organization (WHO) as a critical‐priority pathogen, receiving the highest priority score (84%) among all evaluated pathogens, based on criteria including mortality, healthcare burden, prevalence of resistance, transmissibility, and limited treatment options (Sati et al. 2025; Tacconelli et al. 2018). This ranking reflects its severe clinical impact and alarming ability to produce ESBLs and carbapenemases, which leaves the last‐line antibiotics ineffective. They are widely distributed in nature, including soil, water, plants, animals, and humans. The dissemination of resistance traits is largely driven by horizontal gene transfer, most often via plasmids, which carry many resistance determinants (Asokan et al. 2025b; Navon‐Venezia et al. 2017; Di Pilato et al. 2024). ESBL and carbapenemase production (e.g., NDM‐1, KPC, OXA‐48) often coexist with multi‐drug resistance (MDR) in *K. pneumoniae*, further hindering the effectiveness of existing treatments and deteriorating clinical outcomes (Michodigni et al. 2021; Al‐agamy et al. 2019). It is classified among WHO‐designated “ESKAPE pathogens” that evade antimicrobial therapy and pose a significant global healthcare challenge (Pandey et al. 2021).

Beyond its role as a formidable nosocomial ESKAPE pathogen, *K. pneumoniae* has been isolated from ornamental birds, where it exists as a part of their commensal microbiota, colonizing the gastrointestinal and respiratory tracts (Davies et al. 2016). Although these birds often appear clinically healthy, *K. pneumoniae* is a zoonotic pathogen capable of harboring virulence and AMR determinants (Kekeç et al. 2021; Ahmed et al. 2021; Liu et al. 2025). Over the years, the ornamental bird industry has experienced significant growth, and millions of households worldwide now raise a variety of birds as pets, including budgerigars, parrots, canaries, finches, pigeons, and mynas. Over 50 million ornamental birds are estimated to be kept in captivity worldwide, especially in Europe (Ribeiro et al. 2019). In Bangladesh, regional evidence from Rajshahi City has reported approximately 30 ornamental bird shops trading around 24 bird species (Yasmin et al. 2024). Pet shop owners, dealers, and breeders' profit from ornamental birds, in addition to helping owners relax and feel emotionally better. In Bangladesh and other low‐ and middle‐income countries (LMICs) the popularity of keeping ornamental birds as pets has increased significantly, driven by open markets, informal pet shops, small‐scale breeders, and household‐level trading. Close and frequent contact between birds and people, combined with limited biosecurity, inadequate hygiene practices, and high density of birds in cages, may facilitate the transmission and spread of zoonotic pathogens like *K. pneumoniae*. It can be spread by direct handling of birds, inhaling aerosolized droppings or feather dust, or encountering contaminated cages or equipment (Hasan et al. 2025). Under such conditions, ornamental birds may act as a silent reservoir and dissemination sources of ESBL‐KP and CR‐KP, posing a potential public health risk in LMICs settings. Ornamental birds have received comparatively little attention in AMR studies due to their limited economic importance and fragmented small‐scale rearing systems, despite their close contact with humans and potential role in zoonotic transmission compared with poultry and wild birds. In recent years, a limited number of studies have been conducted, mainly focusing on general bacterial occurrence or overall AMR patterns (Punom et al. 2025). However, there is a notable lack of data specifically addressing ESBL‐producing and carbapenem‐resistant *K. pneumoniae* in ornamental bird populations. The emergence of ESBL‐producing *K. pneumoniae* and carbapenem‐resistant *K. pneumoniae* in this niche poses a critical One Health threat. These resistant bacteria not only jeopardize human, animal, and bird health but also facilitate the environmental dissemination of resistance genes, amplifying the risk of broader ecological contamination. Therefore, a comprehensive molecular characterization of resistance and virulence determinants of these isolates is essential to guide targeted AMR surveillance, strengthen biosecurity measures, and inform effective intervention strategies. This study aimed to investigate the occurrence of *K. pneumoniae* in ornamental birds and their associated environments, and to characterize the isolates in terms of AMR profiles, particularly ESBL and carbapenem resistance, as well as biofilm‐forming ability and virulence gene profiles within a One Health framework.

## 2 Materials and Methods

### 2.1 Ethical Approval

Ethical approval was obtained from the Bangladesh Agricultural University Ethical Committee, Mymensingh‐2202, to conduct all animal experimental procedures. All animals used in the sample collection process were treated in accordance with local animal welfare laws and kept up to date in accordance with accepted practices. The approval number is “AWEEC/BAU/2024(2)/20(a).”

### 2.2 Sample Collection

**Figure 1:**
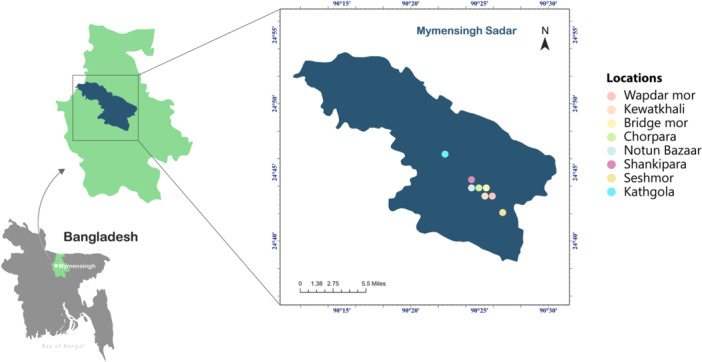
Map of sampling areas.

All the samples were collected from bird owners' houses and bird stores, covering several different areas of Mymensingh Sadar Upazila (24.7460^°^ N, 90.4179^°^ E), including Wapdar mor (24.7349^°^ N,90.4221° E), Kewatkhali (24.7316^°^ N, 90.4163^°^ E), Bridge mor (24.7469^°^ N, 90.4200^°^ E), Chorpara (24.7464° N, 90.4092° E), Notun Bazaar (24.7409° N, 90.3981° E), Shankipara (24.7583° N, 90.3956° E), Seshmor (24.71789° N, 90.44416° E), and Kathgola (24.7753° N, 90.3801° E) (Figure 1). A total of 112 samples were collected for this study, including 56 residential samples that have a long history of raising ornamental birds as well as 56 samples from different pet stores where the bird owners under this study typically purchase birds and feeds. Feces, cage swab, cage water, cage feed, store feed, handler's hand swab, nasal swab, and an air sample of the environment in which the birds were housed were among the eight types collected aseptically. With a minor modification, the settle plate method was used for air sampling (Mbamalu et al. 2015; Ievy et al. 2020). In this case, the MacConkey agar (HiMedia, India) plates were placed in various corners of the rooms for 10 min at a height of one meter above the ground. All the samples, including fresh droppings, were collected using sterile cotton buds and placed in sterile test tubes containing 5 mL of nutrient broth (HiMedia, India). Each sample was assigned a unique identification number and transported to the Department of Microbiology and Hygiene laboratory (24.7245° N, 90.4372° E) at Bangladesh Agricultural University, Mymensingh, maintaining a cold chain. MacConkey agar plates and test tubes containing samples were incubated overnight at 37°C to promote bacterial growth.

### 2.3 Isolation and Molecular Identification of *K. pneumoniae*

Overnight cultured samples were streaked onto the MacConkey agar (HiMedia, India) and incubated for 16–18 h at 37°C. *K. pneumoniae* was primarily identified based on the colony characteristics (large, mucoid, smooth, convex and lactose‐fermenting pink colonies) and subsequently sub‐cultured to obtain pure isolates. These isolates were then subjected to Gram staining and routine biochemical tests (basic five‐sugar fermentation tests, Methyl Red, Voges‐Proskauer, and the indole test) described by Patra et al. (2020). All the isolates were Gram‐negative, rod‐shaped bacteria, positive for sugar fermentation, Voges‐Proskauer and negative for Methyl Red, and the indole test. Genomic DNA was then extracted from pure isolates, using the boiling and chilling method previously described by Fahim et al. (Fahim et al. 2025). For molecular confirmation, genomic DNA were used as the templates for polymerase chain reaction (PCR) with species‐specific primer targeting the *rcs*A gene (Supporting Information S1: Table 1). Three randomly selected PCR products were sent for commercial Sanger sequencing to further validate the findings.

### 2.4 Determination of Biofilm‐Forming Abilities of *K. pneumoniae*

The Congo Red (CR) test was employed to assess the phenotypic biofilm‐forming ability of *K. pneumoniae* isolates (Vuotto et al. 2017; Wang et al. 2020). Biofilm formation was determined by observing the colony morphology on CR agar. Based on colony morphology, isolates producing dry filamentous black colonies were classified as strong biofilm formers, those with pink colonies with a dark core as intermediate and smooth pink colonies as weak or non‐biofilm formers. Biofilm‐associated genes were also detected by PCR to support the phenotypic findings (M. Elbrolosy et al. 2020).

### 2.5 Antibiotic Susceptibility Testing

Antibiotic sensitivity of *K. pneumoniae* isolates was evaluated using the disk diffusion method (Bauer et al. 1966) as per the Clinical and Laboratory Standards Institute (CLSI) guidelines (CLSI 2025). Twelve commonly accessible antibiotics from nine different antibiotic classes were selected, based on World Health Organization's (WHO) AWaRe classification. Isolates exhibiting resistance to at least three antimicrobial classes were classified as MDR (Catalano et al. 2022). The multiple antibiotic resistance (MAR) indices were calculated using the following formula (Krumperman 1983):

$$\text{MAR index}=\frac{\text{The count of antibiotics to which an isolates showed resistance}}{\text{The total number of antibiotics employed in this study}}$$

According to the CLSI 2025 guidelines, isolates showing a zone of inhibition of ≤ 27 mm for cefotaxime and ≤ 25 mm for ceftriaxone were selected for ESBL resistance confirmation tests. Double Disc Synergy Test (DDST) and Phenotypic Confirmatory Disc Diffusion Test (PCDDT) were performed to detect ESBL‐KP (Ajuga et al. 2021; Sarojamma and Ramakrishna 2011). For the DDST, cefotaxime (30 µg) and ceftazidime (30 µg) discs were placed 15 mm apart (center to center) from an amoxicillin‐clavulanic acid (Amoxiclav: 20/10 µg) disc on Mueller‐Hinton agar to screen potential ESBL producers. Potential ESBL producers were identified by observing a distinct shape/size (keyhole effect) with potentiation toward amoxicillin + clavulanate disk. ESBL production was confirmed among potential ESBL‐producing isolates by PCDDT, where ceftazidime (30 µg) disks, alone and in combination with clavulanic acid (30/10 µg), were placed 25 mm apart. An increase of ≥ 5 mm in the inhibition zone diameter for either ceftazidime‐clavulanate combination compared to the ceftazidime alone confirmed ESBL production.

### 2.6 Molecular Detection of Resistance and Virulence Genes of Isolated *K. pneumoniae*

To confirm ESBL production, we performed a PCR screening for beta‐lactamase genes (*bla*_TEM_, *bla*_SHV_, *bla*_CTX‐M_) in the isolates using the primer sets listed in Supporting Information S1: Table 1. Carbapenemase genes (*bla*_NDM,_ *bla*_OXA‐51_) were targeted to detect carbapenem resistance. In addition, virulence genes such as *entB, mrkD, kfu*, and *uge* were also screened using specific primers Supporting Information S1: Table 1. The amplified PCR products were then subjected to visualize in gel electrophoresis using 1.5% agarose gel and confirmed by matching distinct band size.

### 2.7 Bioinformatic Analysis

Partial nucleotide sequences obtained by Sanger sequencing were initially examined using Chromas (version 2.6.6) (http://www.technelysium.com.au/chromas.html) to assess sequence quality, followed by trimming of ambiguous bases and low‐quality sequences using BioEdit (version 7.2.5) (Tom Hall 2011). The species identity of the isolates was confirmed by comparison with reference sequences in the NCBI BLAST database (http://blast.ncbi.nlm.nih.gov/Blast.cgi). Nucleotide sequences were translated into amino acid sequences using ExPASy tools (Gasteiger 2003). Phylogenetic analysis was conducted in MEGA version 12.1 (Stecher et al. 2025).

### 2.8 Statistical Analysis

Data were analyzed using IBM SPSS Statistics (Version 27.0, IBM Corp., Armonk, NY, USA) and Microsoft Excel 2013 (Microsoft Corp., Los Angeles, CA, USA). Chi‐square tests (Z test for proportions) or Fisher's exact test were applied to assess variations in *K. pneumoniae* isolates and virulence genes, with *p* < 0.05 considered statistically significant. The correlations between resistance patterns and virulence genes were evaluated using bivariate analysis.

## 3 Results

### 3.1 Occurrence of *K. pneumoniae*

**Figure 2:**
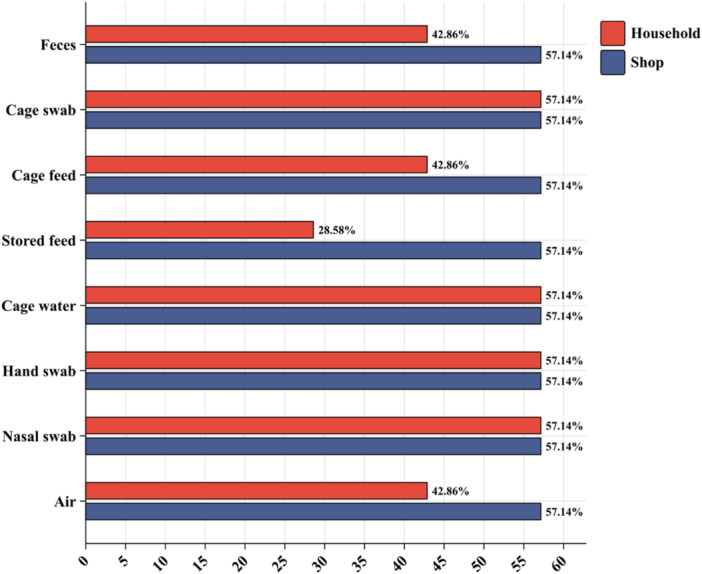
Overall occurrence of *Klebsiella pneumoniae* in various samples.

**Figure 3:**
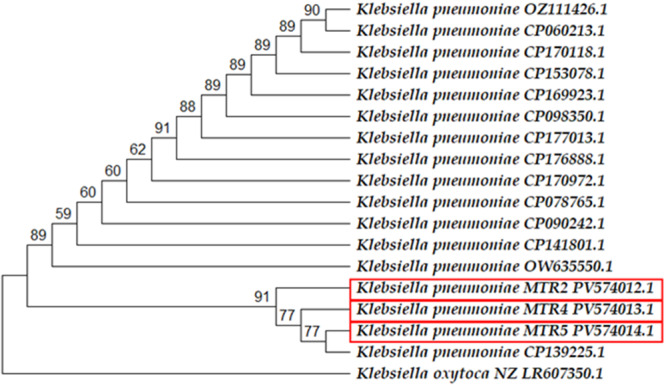
Neighbor‐joining phylogenetic tree constructed in MEGA 12 based on partial *rcsA* gene sequences. Bootstrap analysis was performed with 1000 replicates. The tree is rooted using *Klebsiella oxytoca* (NZ_LR607350.1) as an outgroup. Bootstrap values > 50% are shown at the nodes.

Out of the 112 samples, a total of 59 isolates (52.68%) were confirmed as *K. pneumoniae* through species‐specific PCR (Table 1). A higher occurrence of *K. pneumoniae* was found in shop‐derived samples (57.14%) compared to household‐derived samples (48.21%) (Figure 2). *K. pneumoniae* was further confirmed by Sanger sequencing of three randomly selected isolates (GenBank with accession numbers “**PV574012**,” “**PV574013**,” and “**PV574014**”). These isolates formed a distinct, tightly clustered clade with high bootstrap support, indicating strong genetic relatedness when a neighbor‐joining phylogenetic tree was constructed using MEGA 12 (Figure 3).

**Table 1:** Overall occurrence *Klebsiella pneumoniae* in different samples.

Sl. No.	Sample site	Sample type	No. of Samples Tested	PCR Positive *K. pneumoniae* n (%)	95% CI (%)	Total PCR Positive *K. pneumoniae n* (%)
1	Household (56)	Feces	7	3 (42.86%)	[0.1582–0.7495]	27 (48.21%)
2		Cage swab	7	4 (57.14%)	[0.2505–0.8418]	
3		Cage feed	7	3 (42.86%)	[0.1582–0.7495]	
4		Stored feed	7	2 (28.58%)	[0.0822–0.6411]	
5		Cage water	7	4 (57.14%)	[0.2505–0.8418]	
6		Hand swab	7	4 (57.14%)	[0.2505–0.8418]	
7		Nasal swab	7	4 (57.14%)	[0.2505–0.8418]	
8		Air	7	3 (42.86%)	[0.1582–0.7495]	
1	Shop (56)	Feces	7	4 (57.14%)	[0.2505–0.8418]	32 (57.14%)
2		Cage swab	7	4 (57.14%)	[0.2505–0.8418]	
3		Cage feed	7	4 (57.14%)	[0.2505–0.8418]	
4		Stored feed	7	4 (57.14%)	[0.2505–0.8418]	
5		Cage water	7	4 (57.14%)	[0.2505–0.8418]	
6		Hand swab	7	4 (57.14%)	[0.2505–0.8418]	
7		Nasal swab	7	4 (57.14%)	[0.2505–0.8418]	
8		Air	7	4 (57.14%)	[0.2505–0.8418]	
Total	112				[0.435–0.6168]	59 (52.68%)

### 3.2 Frequency of Biofilm Formation of *K. pneumoniae*

All 59 *K. pneumoniae* isolates were evaluated for biofilm formation using the Congo Red Agar (CRA) method. Among these, 45 isolates (76.28%) were classified as strong biofilm producers, 10 (16.95%) as moderate (intermediate), and 4 (6.78%) as weak/non‐producers (Table 2).

**Table 2:** Occurrence of biofilm‐forming isolates of *Klebsiella pneumoniae* (*n* = 59).

SL no	Biofilm‐forming isolates	Occurrence of biofilm formers (%)	95% CI (%)
1	Strong	45 (76.28%)	[0.6403–0.8531]
2	Intermediate	10 (16.95%)	[0.09476–0.2846]
3	Weak	4 (6.78%)	[0.02668–0.1618]

### 3.3 Virulence Determinants of the Isolated *K. pneumoniae*

Among the 59 *K. pneumoniae* isolates, the *uge* gene was present in all isolates (100%). High occurrence was also observed for *entB* (83.05%) and *mrkD* (81.36%). In contrast, the *kfu* gene was detected in 30.51% of isolates.

Spearman correlation analysis revealed a significant positive association between *entB* and *mrkD* (*r* = 0.364, *p* < 0.01). No significant correlations were observed between *entB* and *kfu* (*r* = 0.005) or between *mrkD* and *kfu* (*r* = 0.128). Correlations involving *uge* could not be computed because the gene exhibited no variability across the isolates, rendering statistical estimation impossible (Supporting Information S1: Table 2).

Regarding biofilm formation, strong biofilm‐forming isolates also showed a markedly higher occurrence of *entB* and *mrkD* (100%) than intermediate and non‐biofilm‐forming isolates, in which both genes were absent, and this association was statistically analyzed using Pearson's Chi‐square test(Table 3). However, *kfu* was predominantly detected in non‐biofilm formers (100%) and less frequently in strong biofilm formers (31.1%), while *uge* was present in all isolates regardless of biofilm phenotype.

**Table 3:** Association of virulence genes and biofilm formation in *Klebsiella pneumoniae* (*n* = 59).

Virulence gene names	Virulence in different degrees of biofilm formation			Total no. of positive Isolates, N^s^ (%) [95% CI]	*p‐*value
	n^s^ (%); No. of Strong biofilm former (*n* = 45)	n^s^ (%); No. (%) of Intermediate biofilm former (*n* = 10)	n^s^ (%); No. (%) of Non‐Biofilm Former (*n* = 4)		
*entB*	45^a^ (100)	4^b^ (40)	0^b^	49 (83.05) (0.7154–0.9052)	< 0.001
*kfu*	14^a^ (31.1)	0^b^	4^c^ (100)	18 (30.51) (0.2025–0.4315)	0.001
*mrkD*	45^a^ (100)	3^b^ (30)	0^b^	48 (81.36) (0.6962–0.8926)	< 0.001
*uge*	45^a^ (100)	10^a^ (100)	4^a^ (100)	59 (100) (0.9389–1)	< 0.001

*Note:* Here, within the variable being evaluated, s = values with different superscripts differ significantly (*p* < 0.05), *n* = no of isolates positive for the category.

Abbreviation: CI = confidence interval.

### 3.4 Antibiotic Resistance Patterns of Isolated *K. pneumoniae*

**Figure 4:**
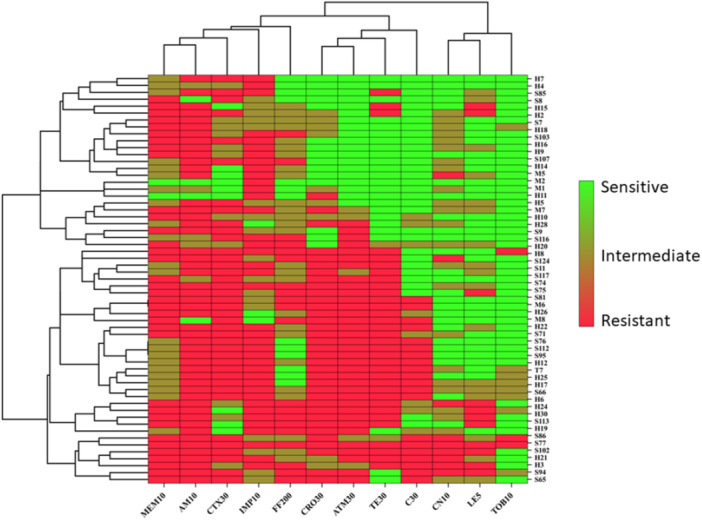
A heatmap showing antibiotic resistance of isolated *Klebsiella pneumoniae*, Abbreviations: AM = Ampicillin, ATM = Aztreonam, CTX = Cefotaxime, CRO = Ceftriaxone, C = Chloramphenicol, CN = Gentamicin, FF = Fosfomycin, IMP = Imipenem, LE = levofloxacin, MEM = Meropenem, TE = Tetracycline, TOB = Tobramycin.

The overall antibiogram study revealed alarmingly high resistance to imipenem (66.10%) and meropenem (59.32%), underscoring the emergence of carbapenem‐resistant strains. Moreover, a high resistance was also observed against cefotaxime (66.10%) and ceftriaxone (59.32%) (Figure 4). These patterns are shown in Figure 4 with a heatmap of *K. pneumoniae* isolates illustrating the distribution of resistant, intermediate, and susceptible isolates within each sample type.

Statistical analysis using Spearman's correlation revealed multiple significant associations among the resistance patterns of the *K. pneumoniae* isolates. Resistance to beta‐lactam antibiotics (Aztreonam, Ceftriaxone, and Cefotaxime) showed strong positive correlations with each other (Aztreonam–Ceftriaxone, *ρ* = 0.725, *p* < 0.01; Aztreonam–Cefotaxime, *ρ* = 0.418, *p* < 0.01; Ceftriaxone–Cefotaxime, *ρ* = 0.314, *p* < 0.05. Aztreonam resistance was also significantly associated with Tetracycline (*ρ* = 0.619, *p* < 0.01), Chloramphenicol (*ρ* = 0.608, *p* < 0.01), and Fosfomycin (*ρ* = 0.353, *p* < 0.01). Levofloxacin resistance correlated positively with Gentamicin (*ρ* = 0.424, *p* < 0.01), Tetracycline (*ρ* = 0.340, *p* < 0.01), Fosfomycin (*ρ* = 0.338, *p* < 0.01), and Meropenem (*ρ* = 0.359, *p* < 0.01), while Tetracycline resistance also correlated with ((*ρ* = 0.590, *p* < 0.01) and Chloramphenicol (*ρ* = 0.454, *p* < 0.01). Fosfomycin resistance showed significant positive correlations with Meropenem (*ρ* = 0.577, *p* < 0.01) and Gentamicin (*ρ* = 0.324, *p* < 0.05). Overall, these findings highlight the presence of multidrug resistance and potential co‐selection of resistance determinants, emphasizing the complexity of AMR patterns in *K. pneumoniae* from ornamental birds and their environments (Supporting Information S1: Table 3).

All *K. pneumoniae* isolates revealed 34 distinct antibiotic‐resistance patterns, with 47 isolates (79.67%) identified as MDR. The MAR index ranged from 0.25 to 1.00, suggesting that these isolates originated from settings with frequent antibiotic use (Supporting Information S1: Table 4).

Moreover, strong biofilm‐forming *K. pneumoniae* isolates revealed significantly higher levels of antibiotic resistance compared to intermediate and non‐biofilm‐forming isolates, particularly to ceftriaxone (32/45, 71.1%), meropenem (31/45, 68.9%), tetracycline (30/45, 66.7%), and chloramphenicol (20/45, 44.4%), and this association was also analyzed using Pearson's Chi‐square test statistically (Table 4).

**Table 4:** Association of antibiotic resistance pattern and biofilm formation in *Klebsiella pneumoniae*.

Antibiotics	Biofilm formation level			Total no. of resistant isolates N^s^ (%) [95% CI]	*p‐*value
	n^s^ (%); No. of strong biofilm former (*N* = 45)	n^s^ (%); No. (%) of intermediate biofilm former (*N* = 10)	n^s^ (%); No. (%) of non‐biofilm former (*N* = 4)		
AM	42^a^ (93.3%)	5^b^ (50%)	4^a,b^ (100%)	51[0.7546–0.9297]	0.001
CN	9^a^ (20%)	0^a^ (0%)	0^a^ (0%)	9[0.08238–0.2652]	0.192
TOB	3^a^ (6.7%)	0^a^ (0%)	0^a^ (0%)	3[0.01744–0.1392]	0.612
C	20^a^ (44.4%)	0^b^ (0%)	0^a,b^ (0%)	20[0.2314–0.4663]	0.009
TE	30^a^ (66.7%)	1^b^ (10%)	2^a,b^ (50%)	33[0.4329–0.6785]	0.005
MEM	31^a^ (68.9%)	3^b^ (30%)	1^a,b^ (25%)	35 [0.4659–0.7091]	0.027
IMP	28^a^ (62.2%)	7^b^ (70%)	4^a,b^ (100%)	39[0.5337–0.7686]	0.104
CRO	32^a^ (71.1%)	1^b^ (10%)	2^a,b^ (50%)	35[0.4659–0.7091]	0.002
CTX	33^a^ (73.3%)	4^b^ (40%)	2^a,b^ (50%)	39[0.5337–0.7686]	0.103
LE	10^a^ (22.2%)	0^a^ (0%)	1^a^ (25%)	11[0.1074–0.3038]	0.249
FF	16^a^ (35.6%)	2^b^ (20%)	2^a,b^ (50%)	20[0.2314–0.4663]	0.502
ATM	30^a^ (66.7%)	3^b^ (30%)	2^a,b^ (50%)	35[0.4659–0.7091]	0.095

*Note:* Here, within the variable being evaluated, s = values with different superscripts differ significantly (*p* < 0.05).

Abbreviatons: AM = Ampicillin, ATM = Aztreonam, C = Chloramphenicol, CI = confidence interval, CN = Gentamicin, CRO = Ceftriaxone, CTX = Cefotaxime, FF = Fosfomycin, IMP = Imipenem, LE = levofloxacin, MEM = Meropenem, N = Total number of isolates sampled, n = no isolates in each biofilm formation category, TOB = Tobramycin, TE = Tetracycline.

The overall occurrence of ESBL‐KP was 64.40%, more prevalent in shop samples (81.25%) than in household samples (44.44%). Several sample types from the shop, including feces, stored feed handler's hand, and nasal swab, and air sample, showed 100% positivity, while household samples showed comparatively lower rates across all categories. Overall, the highest positivity was detected in air (85.72%), stored feed (83.33%), nasal swabs (75%), and feces (71.42%), suggesting multiple possible contamination sources, including fecal shedding, feed handling, and human contact. *K. pneumoniae* was also detected in environmental air samples collected by settle plates, indicating its presence in settling airborne particles within the study settings (Table 5).

**Table 5:** Occurrence of ESBL‐Producing *Klebsiella pneumoniae* in different samples of household and shop origin.

Sample type	Shop (*n* = 32)	Household (*n* = 27)	Total positive	*p‐value*
Feces (7)	4 (100%)	1 (33.33%)	5 (71.42%)	0.143
Cage swab (8)	2 (50%)	1 (25%)	3 (37.5%)	1.00
Cage Feed (7)	2 (50%)	2 (66.67%)	4 (57.14%)	1.00
Stored Feed (6)	4 (100%)	1 (25%)	5 (83.33%)	0.143
Cage Water (8)	2 (50%)	2 (50%)	4 (50%)	1.00
Hand swab (8)	4 (100%)	2 (50%)	5 (62.5%)	0.429
Nasal swab (8)	4 (100%)	1 (25%)	6 (75%)	0.143
Air (7)	4 (100%)	2 (66.67%)	6 (85.72%)	0.429
Total 59	26 (81.25%)	12 (44.44%)	38 (64.40%)	0.002

Phenotypic resistance was observed in 66.10% of isolates for imipenem and 59.32% for meropenem. 59.32% of isolates were resistant to both imipenem and meropenem, as confirmed by PCR targeting carbapenemase genes.

### 3.5 Molecular Detection of Resistance Genes of *K. pneumoniae*

Overall, 38 isolates were identified as ESBL‐KP and 35 as CR‐KP, phenotypically. All of them were subjected to PCR for detection of ESBL‐ and carbapenem‐associated genes. Among these, 32/38 (84.21%) were positive for *bla*_TEM_, 30/38 (78.95%) for *bla*_SHV_, 35/38 (92.10%) for *bla*_CTX‐M_, 12/35 (34.29%) for *bla*_NDM_, and 18/35 (51.42%) for *bla*_OXA‐51,_ confirming the high occurrence of both ESBL and carbapenem resistant gene among phenotypically resistant isolates (Table 6).

**Table 6:** Overall occurrence of resistance genes in the isolated *Klebsiella pneumoniae*.

SL no	Resistance gene	Resistance gene positive %	95% CI (%)
1	*bla*_TEM_	32/38 (84.21%)	(0.6396–0.9476)
2	*bla*_SHV_	30/38 (78.95%)	(0.584–0.9193)
3	*bla*_CTX‐M_	35/38 (92.10%)	(0.699–0.9721)
4	*bla*_NDM_	12/35 (34.29%)	(0.6396–0.9476)
5	*bla*_OXA‐51_	18/35 (51.42%)	(0.699–0.9721)

## 4 Discussion

*Klebsiella pneumoniae* is a critical‐priority pathogen that is becoming a significant public health threat due to its ability to develop multidrug resistance, biofilm formation, and possess a variety of virulence genes. These MDR pathogens can readily be transmitted to humans from ornamental birds and their surrounding environments through cross‐contamination, as they can be a reservoir and possible vectors of *K. pneumoniae*.

In this study, the overall occurrence of *K. pneumoniae* was 52.68% (59/112), with a higher detection rate in shop‐derived samples (32/56, 57.14%) compared to household samples (27/56, 48.21%). Several comparable studies have reported varying prevalence rates depending on sample source and sampling sites. For instance, a study in Egypt reported 17.6% prevalence of *K. pneumoniae* among Budgerigar, Rosy‐faced lovebird, and Red‐rumped parrot (Ahmed et al. 2021), while Samir et al (Samir et al. 2025). recorded a 32% prevalence from nasal swabs of sick parrots in Cairo, Egypt. In India, Singh et al. (Singh et al. 2024) reported a prevalence of 50% in the fecal samples of pigeons. Davies et al. (Davies et al. 2016). documented a lower detection rate in both fecal and respiratory samples of *Psittaciformes* and *Passeriformes* birds in Brazil. Moreover, only a 3.1% prevalence rate was reported from fecal samples of budgerigars and parrots by Kekeç et al (Kekeç et al. 2021). in Turkey. Phylogenetic analysis of selected isolates clustered closely with reference *K. pneumoniae* sequences which were randomly retrieved from NCBI GenBank, confirming their species identity. These isolates formed a distinct sub‐clade with high bootstrap support (77%–91%), indicating close genetic relatedness among the isolates and suggesting a possible shared evolutionary background.

In this study, the observed higher occurrence of *K. pneumoniae* in shop‐derived samples could be due to the higher bird density and turnover, shared cages, frequent human‐bird interactions, and inadequate hygiene management. These factors may provide a favorable condition for bacterial persistence and transmission (Hasan et al. 2025; Islam et al. 2023; Kobuszewska and Wysok 2024). People often visit these shops to purchase ornamental birds or their feed without wearing masks or maintaining personal hygiene, thereby exposing themselves to contaminated fomites, droppings, and cage surfaces. When they take the birds home and feed them, it provides a direct route for cross‐contamination, allowing *K. pneumoniae* to spread from the bird shop into household settings, where it can colonize new hosts and further increase the risk of zoonotic transmission.

In this study, we detected several key virulence genes, *uge* (100%), *entB* (83.05%), *mrkD* (81.36%), and *kfu* (30.51%), in our isolates at a concerning frequency, which, to the best of our knowledge, is the first report among ornamental birds in Bangladesh. These virulence genes were chosen to represent key functional categories relevant to colonization and persistence of *K. pneumoniae* in bird and environmental reservoirs. The *entB* is responsible for enterobactin synthesis and *kfu* is responsible for the iron uptake system, which are critical for survival in nutrient‐limited environments. *mrkD* and *uge* (UDP‐ galacturonate 4‐ epimerase) are involved in type 3 fimbrial adhesin and capsular polysaccharide and lipopolysaccharide biosynthesis respectively, which contribute to biofilm formation and environmental persistence (Soltani et al. 2020; Candan and Aksöz 2015). Although several studies have reported the presence of these virulence genes in poultry, wild, and migratory birds, limited studies have been found on ornamental birds. Davies et al (Davies et al. 2016). documented a high occurrence of *uge* (96.8%), *kfu* (81.2%) and *mrkD* (34.3%) in both fecal and respiratory samples of Psittacine and Passerine birds in Brazil. Daehre et al (Daehre et al. 2018). detected *entB* and *mrkD* from the broiler production chain. Kot et al (Kot et al. 2023). showed a high occurrence of *mrkD* (96.3%) and *entB* (100%) among *K. pneumoniae* in clinical isolates, while *kfu* was detected in 6.4% of isolates, respectively. A statistically significant, moderately strong positive correlation was observed between *entB* and *mrkD* in the bivariate analysis, suggesting a potential genetic linkage or co‐occurrence between these virulence determinants. Together with multidrug and carbapenem resistance, their co‐occurrence points out increased colonization and immune evasion capacity, pointing to a heightened virulence and resistance risk that calls for molecular surveillance in non‐clinical environments (Xia et al. 2022; Mendes et al. 2023).

A notable proportion of strong biofilm formers (76.28%) suggests their strong persistence, antibiotic tolerance, and environmental survival. This aligns with the high prevalence of the *mrkD* gene, indicating a likely association between type 3 fimbriae and robust biofilm formation (M. Elbrolosy et al. 2020). Overall, these traits contribute to the pathogenic potential and multidrug resistance of these isolates, particularly against beta‐lactams and carbapenems (Vintilă et al. 2024; Ali Rahdar et al. 2019). This underscores the critical role of biofilm phenotype in AMR and highlights the importance of targeted interventions.

In this study, the antimicrobial susceptibility profile of *K. pneumoniae* isolates showed an alarming pattern of resistance. A very high proportion of isolates were resistant to access group antibiotics; among them, resistance to tetracycline was observed in 55.94% of the isolates, one of the most commonly used antibiotics in the veterinary field (Authority EFS, Control EC for DP and 2018). Within the watch group, resistance to 3rd‐generation cephalosporins was particularly elevated, with ceftriaxone resistance at 59.32%, whereas cefotaxime resistance was 66.10%. Similarly, aztreonam resistance was also higher (59.32%). 64.40% of isolates were phenotypically ESBL producers. The presence of beta‐lactamase genes, *bla*_TEM_ (84.21%), *bla*_SHV_ (78.95%), and *bla*_CTX‐M_ (92.10%) validates the findings. More concerningly, resistance in the reserve group revealed a similar trend. Overall, 66.10% of isolates were resistant to imipenem, and 59.32% were resistant to meropenem. In this study, *bla*_NDM_ was detected in 34.29% of the *K. pneumoniae* isolates, and *bla*_OXA‐51_ was detected in 51.42%, a remarkably high prevalence that contradicts existing studies, in which this gene was rarely reported in this species (S et al. 2013; Leski et al. 2013). This unexpected finding may indicate unusual dissemination patterns of this resistance gene or novel genetic adaptations, necessitating further genomic characterization to elucidate the underlying mechanisms (Chen et al. 2010). Detailed whole‐genome sequencing and plasmid analysis of the isolated *Klebsiella* spp. could answer such underlying mechanisms.

Such resistance patterns severely impair treatment efficiency and highlight the risk that bird‐related environments serve as hotspots for the selection and dissemination of multidrug‐ and carbapenem‐resistant *K. pneumoniae*, thereby posing a major public health threat within the One Health framework. In this study, 79.67% of *K. pneumoniae* isolates were classified as MDR, with MAR indices ranging from 0.25 to 1.0, indicating considerable antimicrobial pressure in the studied environments.

Although most available studies have focused on captive wild, migratory, or companion birds as well as poultry species, the underlying mechanisms of AMR transmission are comparable to those observed in ornamental birds. The detection of ESBL‐ and CRKP in ornamental birds and their associated environments in the present study aligns with previous reports. A study conducted by Islam et al (Islam et al. 2025). reported high MDR *K. pneumoniae* (63.4%) among wild captive and migratory birds in Sylhet, Bangladesh, with a high *bla*_TEM_ (100%) and *bla*_SHV_ (45.2%) detection rate. Similarly, Ahmed et al (Ahmed et al. 2021). reported high MDR prevalence (90.3%) in *K. pneumoniae* isolates from pet birds in Egypt. An investigation in Brazil, by Davies et (Davies et al. 2016) revealed 22% resistance in tetracycline, 12.5% resistance in chloramphenicol with 25% MDR in psittacine and passerine birds confiscated from illegal trades. Ramatla et al (Ramatla et al. 2024). detected high prevalence of *bla*_CTX‐M_ (50%) in broiler feces in South Africa and Tanni et al (Tanni et al. 2025). observed a prevalence of 86.87% and 31.3% in *bla*_SHV_ and *bla*_TEM,_ respectively in Bangladesh, in another poultry‐based investigation. Several studies have reported the presence of carbapenem‐resistant *K. pneumoniae* in poultry. Fu et al (Fu et al. 2024). detected *bla*_NDM_ (65.56%) in the chicken market in China. However, Chaalal et al (Chaalal et al. 2021). also identified *bla*_NDM_ (20.7%) in poultry carcasses in Western Algeria. Very few studies have reported on *K. pneumoniae* isolation from bird samples exhibiting varying levels of AMR against a range of antimicrobial agents, as documented in studies conducted across diverse regions, including Turkey (Kekeç et al. 2021), China (Wang et al. 2023), Spain (Garcias et al. 2021), Brazil (Davies et al. 2016), and Egypt (Samir et al. 2025).

Spearman's correlation analysis in this study revealed several significant associations among the resistance patterns of *K. pneumoniae* isolates, suggesting possible non‐random clustering of resistance traits and possible shared resistance mechanisms. A higher MAR among the isolates may reflect unregulated antibiotic use, which creates selective pressure favoring resistant strains. The detection of ESBL‐producing and CRKP in both birds and their surrounding environments further suggests cross‐contamination through feed, water or human contact. These findings indicate that ornamental bird settings may function as underrecognized reservoirs of clinically important resistance, emphasizing the need for strengthened One Health surveillance.

## 5 Limitations

This study has some limitations. The settle plate method used for air sampling is a passive and non‐quantitative approach that detects microorganisms present in settling particles. However, its serves as a simple indicator of environmental contamination. In addition, only the Congo Red Agar method was performed, which provided a qualitative assessment of biofilm formation. The crystal violet microtiter assay could not be conducted due to time and resource limitations. We also acknowledge that the study was restricted to a single geographic region, as the primary aim was to highlight the potential role of ornamental bird settings a reservoir of ESBL‐producing carbapenem‐resistant *K. pneumoniae* within One Health context.

## 6 Conclusion

This study demonstrates the presence of biofilm‐forming, ESBL‐producing carbapenem‐resistant *K. pneumoniae* in ornamental birds and their associated environments, indicating their role as potential carriers of MDR *K. pneumoniae*. Their higher presence in environmental samples such as air, cage water, and cage feed indicate improper hygiene and management practices that may favor the persistence and transmission of MDR *K. pneumoniae*. These findings highlight ornamental birds as an important interface in the circulation of MDR *K. pneumoniae* under One Health context. To mitigate these risks, implementation of strict hygiene protocols in pet bird shops, routine surveillance of bird handlers, and strengthened veterinary antibiotic stewardship are strongly recommended. Collectively, these measures are essential to reduce environmental contamination and mitigate potential transmission across animal, human and environmental sectors.

## Author Contributions

**Md Abdullah Evna Hasan:** writing – original draft, methodology, investigation, visualization, software, formal analysis, writing – review and editing, data curation. **Md. Liton Rana:** methodology, writing – review and editing, investigation. **Md. Saiful Islam:** methodology, writing – review and editing. **Sadia Afrin Punom:** writing – review and editing, methodology. **Md Tabeer Hossain Antor:** writing – review and editing. **Diruba Akter Jany:** writing – review and editing. **Naeem Ahammed Ibrahim Fahim:** writing – review and editing. **S M Abu Sama Al Faruquee:** writing – review and editing. **Md. Raisul Islam:** writing – review and editing. **Zuhayr Bakhtiyar:** writing – review and editing. **Anindita Ash Prome:** writing – review and editing. **Md. Monirul Islam:** writing – review and editing. **Saifur Rahman:** writing – review and editing. **Mohammad Ferdousur Rahman Khan:** writing – review and editing, supervision. **Md Tanvir Rahman:** conceptualization, writing – review and editing, resources, project administration, funding acquisition, supervision, validation.

## Ethics Statement

The authors have nothing to report.

## Conflicts of Interest

The authors declare no conflicts of interest.

## Supporting information

Supporting File

## Data Availability

The data that support the findings of this study are available on request from the corresponding author. The data are not publicly available due to privacy or ethical restrictions. Data will be made available on request.

## References

[mbo370348-bib-0001] Ahmed, H. A. , N. F. S. Awad , M. I. Abd El‐Hamid , A. Shaker , R. E. Mohamed , and I. Elsohaby . 2021. “Pet Birds as Potential Reservoirs of Virulent and Antibiotic Resistant Zoonotic Bacteria.” Comparative Immunology, Microbiology and Infectious Diseases 75: 101606. 10.1016/J.CIMID.2020.101606. 33373939

[mbo370348-bib-0002] Ajuga, M. U. , K. Otokunefor , and O. E. Agbagwa . 2021. “Antibiotic Resistance and ESBL Production in Escherichia coli From Various Sources in Aba Metropolis, Nigeria.” Bulletin of the National Research Centre 45: 173. 10.1186/s42269-021-00628-5. 34690489 PMC8524398

[mbo370348-bib-0003] Al‐agamy, M. H. , T. S. El‐mahdy , H. H. Radwan , and L. Poirel . 2019. “Cooccurrence of NDM‐1, ESBL, RmtC, AAC(6′)‐Ib, and QnrB in Clonally Relatedklebsiella Pneumoniaeisolates Together With Coexistence of CMY‐4 and AAC(6′)‐Ib Inenterobacter Cloacaeisolates From Saudi Arabia.” BioMed Research International 2019: 1–7. 10.1155/2019/6736897. PMC6699326 31467906

[mbo370348-bib-0004] Ali Rahdar, H. , E. Shiri Malekabad , A. R. Dadashi , et al. 2019. “Correlation Between Biofilm Formation and Carbapenem Resistance Among Clinical Isolates of Klebsiella pneumoniae.” Ethiopian Journal of Health Sciences 29: 745–750. 10.4314/ejhs.v29i6.11. 31741645 PMC6842719

[mbo370348-bib-0005] Asokan, S. , T. Jacob , J. Jacob , et al. 2025a. “Klebsiella Pneumoniae: A Growing Threat in the Era of Antimicrobial Resistance.” Microbe (Netherlands) 7: 100333. 10.1016/j.microb.2025.100333.

[mbo370348-bib-0006] Asokan, S. , T. Jacob , J. Jacob , A. A. AlSosowaa , and S. Vijayan . 2025b. “Trends in Antimicrobial Susceptibility Patterns of Klebsiella Pneumoniae Isolated From Clinical Samples at a Tertiary Care Hospital in Kerala, India.” Next Res 2: 100654. 10.1016/j.nexres.2025.100654.

[mbo370348-bib-0007] Authority EFS, Control EC for DP and . 2018. “The European Union Summary Report on Antimicrobial Resistance in Zoonotic and Indicator Bacteria From Humans, Animals and Food in 2016.” EFSA Journal 16: e05182. 10.2903/j.efsa.2018.5182. 32625816 PMC7009656

[mbo370348-bib-0008] Bauer, A. W. , W. M. M. Kirby , J. C. Sherris , and M. Turck . 1966. “Antibiotic Susceptibility Testing by a Standardized Single Disk Method.” American Journal of Clinical Pathology 45: 493–496. 10.1093/AJCP/45.4_TS.493. 5325707

[mbo370348-bib-0009] Brisse, S. , and F. Grimont , Prokaryotes PG‐T , 2006 Undefined. 1946. The Genus klebsiella (51, 637. Springer. 10.1007/0-387-30746-X_8.

[mbo370348-bib-0010] Candan, E. D. , and N. Aksöz . 2015. “Klebsiella Pneumoniae: Characteristics of Carbapenem Resistance and Virulence Factors.” Acta Biochimica Polonica 62: 867–874. 10.18388/abp.2015_1148. 26637376

[mbo370348-bib-0011] Catalano, A. , D. Iacopetta , J. Ceramella , et al. 2022. “Multidrug Resistance (MDR): A Widespread Phenomenon in Pharmacological Therapies.” Molecules 27: 616. 10.3390/molecules27030616. 35163878 PMC8839222

[mbo370348-bib-0012] Chaalal, N. , A. Touati , S. Bakour , et al. 2021. “Spread of OXA‐48 and NDM‐1‐Producing Klebsiella pneumoniae ST48 and ST101 in Chicken Meat in Western Algeria.” Microbial Drug Resistance 27: 492–500. 10.1089/MDR.2019.0419. 32208064

[mbo370348-bib-0013] Chen, T. L. , Y. T. Lee , S. C. Kuo , et al. 2010. “Emergence and Distribution of Plasmids Bearing the blaOXA‐51‐ Like Gene With an Upstream ISAba1 in Carbapenem‐Resistant Acinetobacter baumannii Isolates in Taiwan.” Antimicrobial Agents and Chemotherapy 54: 4575–4581. 10.1128/AAC.00764-10. 20713680 PMC2976157

[mbo370348-bib-0014] CLSI . 2025. CLSI M100: Performance Standards for Antimicrobial (35th edition. Clinical and Laboratory.

[mbo370348-bib-0015] Daehre, K. , M. Projahn , A. Friese , T. Semmler , S. Guenther , and U. H. Roesler . 2018. “ESBL‐Producing Klebsiella pneumoniae in the Broiler Production Chain and the First Description of ST3128.” Frontiers in Microbiology 9: 2302. 10.3389/FMICB.2018.02302/FULL. 30337912 PMC6178893

[mbo370348-bib-0016] Davies, Y. M. , M. P. V. Cunha , M. G. X. Oliveira , et al. 2016. “Virulence and Antimicrobial Resistance of Klebsiella pneumoniae Isolated From Passerine and Psittacine Birds.” Avian Pathology 45: 194–201. 10.1080/03079457.2016.1142066. 26813537

[mbo370348-bib-0017] Fahim, N. A. I. , M. L. Rana , M. A. E. Hasan , et al. 2025. “Biofilms and Antibiotic Resistance Profile of Enterococcus Faecalis in Selected Dairy Cattle Farm Environments in Bangladesh.” PLoS ONE 20: e0323667. 10.1371/journal.pone.0323667. 40388390 PMC12087997

[mbo370348-bib-0018] Fu, B. , J. Xu , D. Yin , et al. 2024. “Transmission of blaNDM in Enterobacteriaceae Among Animals, Food and Human.” Emerging Microbes & Infections 13, no. 1: e2337678. 10.1080/22221751.2024.2337678;CTYPE:STRING:JOURNAL. PMC11034458 38629492

[mbo370348-bib-0019] Garcias, B. , L. Aguirre , C. Seminati , et al. 2021. “Extended‐Spectrum β‐Lactam Resistant *Klebsiella pneumoniae* and Escherichia coli in Wild European Hedgehogs (Erinaceus europeus) Living in Populated Areas.” Animals: An Open Access Journal From MDPI 11: 2837. 10.3390/ani11102837. 34679858 PMC8532684

[mbo370348-bib-0020] Gasteiger, E. 2003. “ExPASy: The Proteomics Server for In‐Depth Protein Knowledge and Analysis.” Nucleic Acids Research 31: 3784–3788. 10.1093/nar/gkg563. 12824418 PMC168970

[mbo370348-bib-0021] El Haddad, L. , C. P. Harb , M. A. Gebara , M. A. Stibich , and R. F. Chemaly . 2019. “A Systematic and Critical Review of Bacteriophage Therapy Against Multidrug‐Resistant ESKAPE Organisms in Humans.” Clinical Infectious Diseases 69: 167–178. 10.1093/cid/ciy947. 30395179

[mbo370348-bib-0022] Hasan, M. A. E. , M. S. Islam , N. A. I. Fahim , et al. 2025. “Impact of Open Markets on Zoonotic Threats and Antimicrobial Resistance: A One Health Concern.” One Health 21: 101228. 10.1016/j.onehlt.2025.101228. 41127627 PMC12538495

[mbo370348-bib-0023] Hindieh, P. , J. Yaghi , J. C. Assaf , et al. 2025. “Emerging Multimodal Strategies for Bacterial Biofilm Eradication: A Comprehensive Review.” Microorganisms 13: 2796. 10.3390/microorganisms13122796. 41471999 PMC12735920

[mbo370348-bib-0024] Ievy, S. , M. S. Islam , M. A. Sobur , et al. 2020. “Molecular Detection of Avian Pathogenic Escherichia coli (Apec) for the First Time in Layer Farms in Bangladesh and Their Antibiotic Resistance Patterns.” Microorganisms 8: 1021. 10.3390/microorganisms8071021. 32660167 PMC7409187

[mbo370348-bib-0025] Islam, A. , M. Z. Rahman , M. M. Hassan , J. H. Epstein , and M. Klaassen . 2023. “Determinants for the Presence of Avian Influenza Virus in Live Bird Markets in Bangladesh: Towards an Easy Fix of a Looming One Health Issue.” One Health 17: 100643. 10.1016/j.onehlt.2023.100643. 38024264 PMC10665153

[mbo370348-bib-0026] Islam, M. M. , M. B. Uddin , H. Hossain , et al. 2025. “Molecular Identification and Antimicrobial Resistance Characteristics of Extended‐Spectrum Beta‐Lactamase Producing *Klebsiella pneumoniae* Isolated From Captive Wild and Migratory Birds.” Veterinary Sciences 12: 556. 10.3390/vetsci12060556. 40559793 PMC12197686

[mbo370348-bib-0027] Kekeç, I. , B. Halaç , B. Başaran , and S. Ak . 2021. “Determination of the Presence of *Klebsiella pneumoniae* and Phenotypic Antibiotic Resistance Profiles in Budgerigars and Parrots.” Journal of Anatolian Environmental and Animal Sciences 6: 372–375. 10.35229/jaes.938912.

[mbo370348-bib-0028] Keynan, Y. , and E. Rubinstein . 2007. “The Changing Face of *Klebsiella pneumoniae* Infections in the Community.” International Journal of Antimicrobial Agents 30: 385–389. 10.1016/j.ijantimicag.2007.06.019. 17716872

[mbo370348-bib-0029] Kobuszewska, A. , and B. Wysok . 2024. “Pathogenic Bacteria in Free‐Living Birds, and Its Public Health Significance.” Animals: An Open Access Journal From MDPI 14: 968. 10.3390/ani14060968. 38540066 PMC10967383

[mbo370348-bib-0030] Koo, H. , R. N. Allan , R. P. Howlin , P. Stoodley , and L. Hall‐Stoodley . 2017. “Targeting Microbial Biofilms: Current and Prospective Therapeutic Strategies.” Nature Reviews Microbiology 15: 740–755. 10.1038/nrmicro.2017.99. 28944770 PMC5685531

[mbo370348-bib-0031] Kot, B. , M. Piechota , P. Szweda , et al. 2023. “Virulence Analysis and Antibiotic Resistance of *Klebsiella pneumoniae* Isolates From Hospitalised Patients in Poland.” Scientific Reports 13: 4448. 10.1038/s41598-023-31086-w. 36932105 PMC10023695

[mbo370348-bib-0032] Krumperman, P. H. 1983. “Multiple Antibiotic Resistance Indexing of *Escherichia coli* to Identify High‐Risk Sources of Fecal Contamination of Foods.” Applied and Environmental Microbiology 46: 165–170. 10.1128/AEM.46.1.165-170.1983;WGROUP:STRING:PUBLICATION. 6351743 PMC239283

[mbo370348-bib-0033] Leski, T. A. , U. Bangura , D. H. Jimmy , et al. 2013. “Identification of blaOXA‐51‐like, blaOXA‐58, bla DIM‐1, and blaVIM Carbapenemase Genes in Hospital Enterobacteriaceae Isolates From Sierra Leone.” Journal of Clinical Microbiology 51: 2435–2438. 10.1128/JCM.00832-13/SUPPL_FILE/ZJM999092634SO2.PDF. 23658259 PMC3697688

[mbo370348-bib-0034] Liu, H. C. , H. L. Tang , C. S. Chiou , et al. 2025. “Prevalence and Virulence Profiles of *Klebsiella pneumoniae* Isolated From Different Animals.” Veterinary Medicine and Science 11: e70243. 10.1002/vms3.70243. 39969166 PMC11837280

[mbo370348-bib-0035] Mbamalu, O. , R. Uebel , and B. Meki . 2015. “Control of Airborne Microbes in a Poultry Setting Using Dioxy MP 14.” Revista Brasileira de Ciência Avícola 17: 77–86. 10.1590/1516-635x170177-86.

[mbo370348-bib-0036] M. Elbrolosy, A. , N. A. Eissa , N. A. al‐Rajhy , E. El‐Sayed a. el‐Mahdy , and R. G. Mostafa . 2020. “MrkD Gene as a Regulator of Biofilm Formation With Correlation to Antibiotic Resistance Among Clinical *Klebsiella pneumoniae* Isolates From Menoufia University Hospitals.” Egyptian Journal of Medical Microbiology 29: 137–144. 10.51429/EJMM29318.

[mbo370348-bib-0037] Mendes, G. , M. L. Santos , J. F. Ramalho , A. Duarte , and C. Caneiras . 2023. “Virulence Factors in Carbapenem‐Resistant Hypervirulent *Klebsiella pneumoniae* .” Frontiers in Microbiology 14: 1325077. 10.3389/fmicb.2023.1325077. 38098668 PMC10720631

[mbo370348-bib-0038] Michodigni, N. F. , A. Nyachieo , J. K. Akhwale , G. Magoma , and A. N. Kimang'a . 2021. “Molecular Identification of Co‐Existence of Carbapenemase and Extended‐Spectrum β‐Lactamase Genes in *Klebsiella pneumoniae* Clinical Isolates, and Their Phylogenetic Patterns in Kenya.” Advances in Microbiology 11: 399–415. 10.4236/aim.2021.118030.

[mbo370348-bib-0039] Murray, C. J. L. , K. S. Ikuta , F. Sharara , et al. 2022. “Global Burden of Bacterial Antimicrobial Resistance in 2019: A Systematic Analysis.” Lancet 399: 629–655. 10.1016/S0140-6736(21)02724-0. 35065702 PMC8841637

[mbo370348-bib-0040] Navon‐Venezia, S. , K. Kondratyeva , and A. Carattoli . 2017. “Klebsiella Pneumoniae: A Major Worldwide Source and Shuttle for Antibiotic Resistance.” FEMS Microbiology Reviews 41: 252–275. 10.1093/femsre/fux013. 28521338

[mbo370348-bib-0041] Pandey, R. , S. K. Mishra , and A. Shrestha . 2021. “Characterisation of Eskape Pathogens With Special Reference to Multidrug Resistance and Biofilm Production in a Nepalese Hospital.” Infection and Drug Resistance 14: 2201–2212. 10.2147/IDR.S306688. 34163185 PMC8214009

[mbo370348-bib-0042] Patra, J. K. , G. Das , S. K. Das , and H. Thatoi . 2020. Isolation, Culture, and Biochemical Characterization of Microbes, 83–133. Springer. 10.1007/978-981-15-6252-5_4.

[mbo370348-bib-0043] Di Pilato, V. , S. Pollini , V. Miriagou , G. M. Rossolini , and M. M. D'Andrea . 2024. “Carbapenem‐Resistant Klebsiella Pneumoniae: The Role of Plasmids in Emergence, Dissemination, and Evolution of a Major Clinical Challenge.” Expert Review of Anti‐Infective Therapy 22: 25–43. 10.1080/14787210.2024.2305854. 38236906

[mbo370348-bib-0044] Punom, S. A. , M. S. Islam , N. A. I. Fahim , et al. 2025. “Ornamental Birds: Hidden Carriers of Potentially Virulent and Antimicrobial‐Resistant *Enterococcus faecalis* in Bangladesh.” Microbiology Spectrum 13: e01974‐25. 10.1128/SPECTRUM.01974-25. PMC12671201 41222237

[mbo370348-bib-0045] Ramatla, T. , P. Mokgokong , K. Lekota , and O. Thekisoe . 2024. “Antimicrobial Resistance Profiles of Pseudomonas Aeruginosa, *Escherichia coli* and *Klebsiella pneumoniae* Strains Isolated From Broiler Chickens.” Food Microbiology 120: 104476. 10.1016/J.FM.2024.104476. 38431322

[mbo370348-bib-0046] Ribeiro, J. , L. Reino , S. Schindler , et al. 2019. “Trends in Legal and Illegal Trade of Wild Birds: A Global Assessment Based on Expert Knowledge.” Biodiversity and Conservation 28: 3343–3369. 10.1007/s10531-019-01825-5.

[mbo370348-bib-0047] S, B. , A. Z , O. O , et al. 2013. “Detection of Oxa‐51 Carbapenemase Gene in *Klebsiella pneumoniae*: A Case Report and a New Dimension on Carbapenemase Resistance.” Journal of Molecular and Genetic Medicine 07: 63. 10.4172/1747-0862.1000063.

[mbo370348-bib-0048] Samir, A. , T. Mosallam , H. Aboul‐Ella , et al. 2025. “Zoonotic Relevance of Multidrug‐Resistant Bacteria in Parrots With Respiratory Illness.” Veterinary Research Communications 49: 194. 10.1007/s11259-025-10752-6. 40338404 PMC12062053

[mbo370348-bib-0049] Sarojamma, V. , and V. Ramakrishna . 2011. “Prevalence of ESBL‐Producing *Klebsiella pneumoniae* Isolates in Tertiary Care Hospital.” ISRN Microbiology 2011: 1–5. 10.5402/2011/318348. PMC3658478 23724303

[mbo370348-bib-0050] Sati, H. , E. Carrara , A. Savoldi , et al. 2025. “The WHO Bacterial Priority Pathogens List 2024: A Prioritisation Study to Guide Research, Development, and Public Health Strategies Against Antimicrobial Resistance.” Lancet Infectious Diseases 25: 1033–1043. 10.1016/S1473-3099(25)00118-5. 40245910 PMC12367593

[mbo370348-bib-0051] Singh, S. , P. Sharma , M. Kriti , et al. 2024. “Urban‐Dwelling Pigeons as Reservoirs of Multidrug‐Resistant Enteric Bacteria and.” Antibiotic Resistance Genes. 10.2139/SSRN.5025982.

[mbo370348-bib-0052] Soltani, E. , A. Hasani , M. Ahangarzadeh Rezaee , et al. 2020. “Virulence Characterization of *Klebsiella pneumoniae* and Its Relation With ESBL and AMPC Beta‐Lactamase Associated Resistance.” Iranian Journal of Microbiology 12: 98–106. 10.18502/ijm.v12i2.2613. 32494343 PMC7244816

[mbo370348-bib-0053] Stecher, G. , M. Suleski , Q. Tao , K. Tamura , and S. Kumar . 2025. “MEGA 12. 1: Cross‐Platform Release for macOS and Linux Operating Systems.” Journal of Molecular Evolution 94, no. 1: 14–18. 41247409 10.1007/s00239-025-10287-z PMC13227079

[mbo370348-bib-0054] Tacconelli, E. , E. Carrara , A. Savoldi , et al. Discovery, Research, And Development Of New Antibiotics: The WHO Priority List of Antibiotic‐resistant Bacteria and Tuberculosis 2018;18:2018. 10.1016/S1473-3099(17)30753-3 29276051

[mbo370348-bib-0055] Tanni, F. Y. , M. S. Rahman Chowdhury , H. Hossain , et al. 2025. “Prevalence and Antimicrobial Resistance of Extended Spectrum Beta‐Lactamase (ESBL) Producing Klebsiella Spp. in Poultry Meat.” Heliyon 11: e41748. 10.1016/J.HELIYON.2025.E41748/ASSET/7CB0BEE1-23FE-4E6C-BFD7-ED257342EC41/MAIN.ASSETS/GR1.JPG. 39866402 PMC11761286

[mbo370348-bib-0056] Tom Hall . 2011. “Non‐Motor Symptoms and Parkinsonism.” Canadian Journal of Neurological Sciences/Journal Canadien des Sciences Neurologiques 40: 1–2. 10.1017/S0317167100012865. 23250119

[mbo370348-bib-0057] Vintilă, C. , R. L. Coșeriu , A. D. Mare , et al. 2024. “Biofilm Formation and Antibiotic Resistance Profiles in Carbapenemase‐Producing Gram‐Negative Rods—A Comparative Analysis Between Screening and Pathological Isolates.” Antibiotics (USSR) 13: 687. 10.3390/antibiotics13080687. PMC11350898 39199988

[mbo370348-bib-0058] Vuotto, C. , F. Longo , C. Pascolini , et al. 2017. “Biofilm Formation and Antibiotic Resistance in *Klebsiella pneumoniae* Urinary Strains.” Journal of Applied Microbiology 123: 1003–1018. 10.1111/jam.13533. 28731269

[mbo370348-bib-0059] Wang, G. , G. Zhao , X. Chao , L. Xie , and H. Wang . 2020. “The Characteristic of Virulence, Biofilm and Antibiotic Resistance of *Klebsiella pneumoniae* .” International Journal of Environmental Research and Public Health 2020, Vol 17, Page 6278 17: 6278. 10.3390/IJERPH17176278. 32872324 PMC7503635

[mbo370348-bib-0060] Wang, X. , J. Zhao , F. Ji , et al. 2023. “Genomic Characteristics and Molecular Epidemiology of Multidrug‐Resistant *Klebsiella pneumoniae* Strains Carried by Wild Birds.” Microbiology Spectrum 11: e02691‐22. 10.1128/SPECTRUM.02691-22/SUPPL_FILE/REVIEWER-COMMENTS.PDF. 36840587 PMC10101063

[mbo370348-bib-0061] Xia, P. , M. Yi , Y. Yuan , et al. 2022. “Coexistence of Multidrug Resistance and Virulence in a Single Conjugative Plasmid From a Hypervirulent *Klebsiella pneumoniae* Isolate of Sequence Type 25.” mSphere 7: e00477‐22. 10.1128/msphere.00477-22. 36472445 PMC9769751

[mbo370348-bib-0062] Yasmin, N. , M. A. Khan , and M. G. Mortuza . 2024. “An Account of Ornamental Bird Status and Species Assemblage in the Bird Shop of Rajshahi City Corporation, Bangladesh.” International Journal of Environment, Agriculture and Biotechnology 9: 083–087. 10.22161/ijeab.93.9.

