# Supplementary material for: Detection and Characterization of ESBL‐Producing and Carbapenem‐Resistant Klebsiella pneumoniae in Ornamental Birds and Their Surrounding Environments
Source: Microbiologyopen. 2026 Jun 23;15(3):e70348. doi: 10.1002/mbo3.70348 (PMC13290651; doi:10.1002/mbo3.70348)
Supplement: Supplementary file 1 — Supporting File [file MBO3-15-e70348-s001.docx]

**Supplementary Table 1:** Oligonucleotide sequences, targeted genes, amplicon size, and Annealing temperature of used primers

| **Target gene** | **Sequence (5′-3′)** | **Amplicon size (bp)** | **Annealing temperature** | **References** |
| --- | --- | --- | --- | --- |
| *rcsA* | F: GGATATCTGACCAGTCGG  R: GGGTTTTGCGTAATGATCTG | 176 bp | 55°C, 30 sec | (Dong et al., 2015) |
| *uge* | F: TCT TCA CGC CTT CCT TCA CT  R: GAT CAT CCG GTC TCC CTG TA | 535 bp | 53°C, 30 sec | (Khalefa et al., 2025) |
| *mrkD* | F: AAGCTATCGCTGTACTTCCGGCA  R: GGCGTTGGCGCTCAGATAGG | 340 bp | 62°C, 30 sec | (Albasha et al., 2020) |
| *entB* | F: GTCAACTGGGCCTTTGAGCCGTC  R: TATGGGCGTAAACGCCGGTGAT | 400 bp |  |  |
| *kfu* | F: GGCCTTTGTCCAGAGCTACG  R: GGGTCTGGCGCAGAGTATGC | 638 bp |  |  |
| *bla*_TEM_ | F: CATTTCCGTGTCGCCCTTAT  R: TCCATAGTTGCCTGACTCCC | 793 bp | 55°C, 1 min | (Walker et al., 2001) |
| *bla*_SHV_ | F: GGGTTATTCTTATTTGTCGC  R: TTAGCGTTGCCAGTGCTC | 615 bp | 59°C, 1 min | (Feizabadi et al., 2010) |
| *bla*_CTX-M_ | F: AACCGTCACGCTGTTGTTAG  R: TTGAGGCTGGGTGAAGTAAG | 766 bp | 55°C, 1 min | (Ehsan et al., 2023) |
| *bla*_NDM_ | F: GGTTTGGCGATCTGGTTTTC  R: CGGAATGGCTCATCACGATC | 521 bp | 59°C, 1 min | (Albasha et al., 2020) |
| *bla*_OXA-51_ | F: TAATGCTTTGATCGGCCTTG  R: TGGATTGCACTTCATCTTGG | 353 bp | 57°C, 45 sec | (Woodford et al., 2006) |

**Supplementary Table 2: Spearman’s correlation coefficient in the virulence gene of the isolated** ***Klebsiella pneumoniae* isolates**

| **Correlations** | | | | | |
| --- | --- | --- | --- | --- | --- |
|  |  | *entB* | *mrkD* | *kfu* | *uge* |
| *entB* | Pearson Correlation | 1 | .364** | 0.005 | .b |
| *mrkD* | Pearson Correlation | .364** | 1 | 0.128 | .b |
| *kfu* | Pearson Correlation | 0.005 | 0.128 | 1 | .b |
| *uge* | Pearson Correlation | .b | .b | .b | 1 |
| ** Correlation is significant at the 0.01 level (2-tailed). | | | | |  |
| b Cannot be computed because at least one of the variables is constant. | | | | | |

**Supplementary Table 3: Spearman’s correlation coefficient in *Klebsiella pneumoniae* isolates**

| **Correlations** | | | | | | | | | | | | | |
| --- | --- | --- | --- | --- | --- | --- | --- | --- | --- | --- | --- | --- | --- |
|  |  | LE5 | AM10 | ATM30 | TOB10 | CN10 | TE30 | FF200 | CRO30 | MEM 10 | C30 | IMP 10 | CTX30 |
| LE5 | PC | 1 | 0.221 | 0.13 | 0.155 | .424** | .340** | .338** | 0.148 | .359** | 0.079 | 0.045 | -0.083 |
| AM10 | PC | 0.221 | 1 | 0.217 | 0.177 | .299* | 0.251 | 0.229 | 0.229 | .287* | 0.187 | 0.101 | 0.23 |
| ATM30 | PC | 0.13 | 0.217 | 1 | 0.216 | 0.023 | .619** | .353** | .725** | 0.199 | .608** | -0.088 | .418** |
| TOB10 | PC | 0.155 | 0.177 | 0.216 | 1 | 0.234 | 0.193 | 0.058 | .307* | 0.067 | .336** | 0.08 | 0.166 |
| CN10 | PC | .424** | .299* | 0.023 | 0.234 | 1 | -0.055 | .324* | 0.085 | 0.255 | 0.146 | 0.04 | -0.058 |
| TE30 | PC | .340** | 0.251 | .619** | 0.193 | -0.055 | 1 | 0.117 | .590** | 0.186 | .454** | 0.045 | .338** |
| FF200 | PC | .338** | 0.229 | .353** | 0.058 | .324* | 0.117 | 1 | 0.205 | .577** | 0.008 | -0.168 | -0.017 |
| CRO30 | PC | 0.148 | 0.229 | .725** | .307* | 0.085 | .590** | 0.205 | 1 | 0.099 | .538** | -0.143 | .314* |
| MEM 10 | PC | .359** | .287* | 0.199 | 0.067 | 0.255 | 0.186 | .577** | 0.099 | 1 | 0.038 | -.370** | 0.171 |
| C30 | PC | 0.079 | 0.187 | .608** | .336** | 0.146 | .454** | 0.008 | .538** | 0.038 | 1 | -0.073 | .365** |
| IMP10 | PC | 0.045 | 0.101 | -0.088 | 0.08 | 0.04 | 0.045 | -0.168 | -0.143 | -.370** | -0.073 | 1 | -0.246 |
| CTX30 | PC | -0.083 | 0.23 | .418** | 0.166 | -0.058 | .338** | -0.017 | .314* | 0.171 | .365** | -0.246 | 1 |
| ** Correlation is significant at the 0.01 level (2-tailed). | | | | | |  |  |  |  |  |  |  |  |
| * Correlation is significant at the 0.05 level (2-tailed). | | | | | |  |  |  |  |  |  |  |  |

**Legends, PC=Pearson correlation; LE= levofloxacin; AM= Ampicillin; ATM= Aztreonam; TOB= Tobramycin; CN= Gentamicin; TE= Tetracycline; FF= Fosfomycin; CRO= Ceftriaxone; MEM= Meropenem; C= Chloramphenicol; IMP= Imipenem; CTX= Cefotaxime.**

**Supplementary Table 4: Occurrence of multi-drug resistance (MDR) and multiple-antibiotic resistance (MAR) in the isolated *Klebsiella pneumoniae***

| SL No | Antibiotic Resistance Pattern | No. of antibiotics (classes) | No. of isolates | Overall, MDR isolates % | MAR index |
| --- | --- | --- | --- | --- | --- |
| 1 | LE, AM, ATM, TE, FF, CRO, MEM, IMP | 8(7) | 3 | 47/59 (79.67%) | 0.67 |
| 2 | AM, FF, MEM, IMP | 4(3) | 1 |  | 0.33 |
| 3 | LE, AM, ATM, TOB, CN, TE, FF, CRO, MEM, C, IMP, CTX | 12(9) | 1 |  | 1 |
| 4 | AM, ATM, FF, CRO, IMP | 5(5) | 1 |  | 0.42 |
| 5 | AM, TE, CRO, IMP, CTX | 5(4) | 1 |  | 0.42 |
| 6 | AM, TE, IMP, CTX | 4 (4) | 2 |  | 0.33 |
| 7 | AM, CN, IMP | 3(3) | 1 |  | 0.25 |
| 8 | ATM, FF, IMP, CTX | 4(4) | 1 |  | 0.33 |
| 9 | AM, IMP, CTX | 3(3) | 1 |  | 0.25 |
| 10 | ATM, FF, MEM, IMP | 4(3) | 1 |  | 0.33 |
| 11 | LE, AM, TE, MEM | 4(4) | 1 |  | 0.33 |
| 12 | AM, MEM, IMP, CTX | 4(3) | 1 |  | 0.33 |
| 13 | LE, AM, TE, MEM, CTX | 5(5) | 1 |  | 0.42 |
| 14 | AM, CRO, MEM, IMP, CTX | 5(4) | 1 |  | 0.42 |
| 15 | AM, TOB, CN, CRO, MEM, C, CTX | 7(6) | 1 |  | 0.59 |
| 16 | AM, ATM, CRO, CTX | 4(3) | 1 |  | 0.33 |
| 17 | LE, AM, ATM, CN, FF, CRO, MEM, C, CTX | 9(8) | 1 |  | 0.75 |
| 18 | AM, ATM, TE, FF, CRO, MEM, IMP, CTX | 8(6) | 1 |  | 0.67 |
| 19 | AM, ATM, TE, CRO, IMP, CTX | 6(5) | 1 |  | 0.5 |
| 20 | AM, ATM, FF, CRO, MEM, C, CTX | 7(7) | 1 |  | 0.59 |
| 21 | AM, TOB, FF, MEM, C | 5(5) | 1 |  | 0.42 |
| 22 | AM, ATM, TE, CRO, MEM, IMP, CTX | 7(5) | 1 |  | 0.59 |
| 23 | AM, ATM, TE, CRO, C, IMP, CTX | 8(6) | 10 |  | 0.67 |
| 24 | LE, AM, ATM, TE, FF, CRO, MEM, CTX | 8(7) | 1 |  | 0.67 |
| 25 | LE, AM, ATM, CN, TE, CRO, MEM, C, CTX | 9(8) | 1 |  | 0.75 |
| 26 | ATM, TE, FF, CRO, MEM, C, CTX | 7(6) | 1 |  | 0.59 |
| 27 | AM, ATM, TE, FF, CRO, MEM, C, CTX | 8(7) | 2 |  | 0.67 |
| 28 | LE, AM, ATM, CN, TE, FF, CRO, MEM, IMP | 9(8) | 1 |  | 0.75 |
| 29 | AM, ATM, TE, CRO, MEM, CTX | 6(5) | 1 |  | 0.5 |
| 30 | AM, ATM, CN, TE, MEM, C, IMP, CTX | 8(7) | 1 |  | 0.67 |
| 31 | LE, AM, CN, TE, FF, MEM, C, IMP | 8(7) | 1 |  | 0.67 |
| 32 | AM, ATM, MEM, CTX | 4(4) | 1 |  | 0.33 |
| 33 | AM, ATM, CN, TE, FF, CRO, MEM, CTX | 8(7) | 1 |  | 0.67 |
| 34 | AM, ATM, TOB, TE, FF, CRO, MEM, C, IMP, CTX | 10(8) | 1 |  | 0.83 |

**References:**

Albasha, A. M., Osman, E. H., Abd-Alhalim, S., Alshaib, E. F., Al-Hassan, L., & Altayb, H. N. (2020). Detection of several carbapenems resistant and virulence genes in classical and hyper-virulent strains of Klebsiella pneumoniae isolated from hospitalized neonates and adults in Khartoum. *BMC Research Notes*, *13*(1), 1–7. https://doi.org/10.1186/s13104-020-05157-4

Dong, D., Liu, W., Li, H., Wang, Y., Li, X., Zou, D., Yang, Z., Huang, S., Zhou, D., Huang, L., & Yuan, J. (2015). Survey and rapid detection of Klebsiella pneumoniae in clinical samples targeting the rcsA gene in Beijing, China. *Frontiers in Microbiology*, *6*(MAY). https://doi.org/10.3389/FMICB.2015.00519/FULL

Ehsan, B., Haque, A., Qasim, M., Ali, A., & Sarwar, Y. (2023). High prevalence of extensively drug resistant and extended spectrum beta lactamases (ESBLs) producing uropathogenic Escherichia coli isolated from Faisalabad, Pakistan. *World Journal of Microbiology and Biotechnology*, *39*(5), 1–13. https://doi.org/10.1007/s11274-023-03565-9

Feizabadi, M. M., Delfani, S., Raji, N., Majnooni, A., Aligholi, M., Shahcheraghi, F., Parvin, M., & Yadegarinia, D. (2010). Distribution of blaTEM, blaSHV, blaCTX-M genes among clinical isolates of Klebsiella pneumoniae at Labbafinejad Hospital, Tehran, Iran. *Microbial Drug Resistance*, *16*(1), 49–53. https://doi.org/10.1089/mdr.2009.0096

Khalefa, H. S., Arafa, A. A., Hamza, D., El-Razik, K. A. A., & Ahmed, Z. (2025). Emerging biofilm formation and disinfectant susceptibility of ESBL-producing Klebsiella pneumoniae. *Scientific Reports*, *15*(1), 1599. https://doi.org/10.1038/s41598-024-84149-x

Walker, R. A., Lindsay, E., Woodward, M. J., Ward, L. R., & Threlfall, E. J. (2001). Variation in clonality and antibiotic-resistance genes among multiresistant Salmonella enterica serotype typhimurium phage-type U302 (MR U302) from humans, animals, and foods. *Microbial Drug Resistance*, *7*(1), 13–21. https://doi.org/10.1089/107662901750152701

Woodford, N., Ellington, M. J., Coelho, J. M., Turton, J. F., Ward, M. E., Brown, S., Amyes, S. G. B., & Livermore, D. M. (2006). Multiplex PCR for genes encoding prevalent OXA carbapenemases in Acinetobacter spp. *International Journal of Antimicrobial Agents*, *27*(4), 351–353. https://doi.org/10.1016/j.ijantimicag.2006.01.004
